# Digital Self-Interference Cancellation Strategies for In-Band Full-Duplex: Methods and Comparisons

**DOI:** 10.3390/s25226835

**Published:** 2025-11-08

**Authors:** Amirmohammad Shahghasi, Gabriel Montoro, Pere L. Gilabert

**Affiliations:** Department of Signal Theory and Communications, Universitat Politècnica de Catalunya (UPC)—Barcelona Tech, 08034 Barcelona, Spain; amirmohammad.shahghasi@upc.edu (A.S.);

**Keywords:** in-band full-duplex, digital self-interference cancellation, generalized memory polynomial, Itô–hermite polynomials, digital predistortion, least squares, least mean squares, fast Kalman, phase-normalized neural networks

## Abstract

In-band full-duplex (IBFD) communication systems offer a promising means of improving spectral efficiency by enabling simultaneous transmission and reception on the same frequency channel. Despite this advantage, self-interference (SI) remains a major challenge to their practical deployment. Among the different SI cancellation (SIC) techniques, this paper focuses on digital SIC methodologies tailored for multiple-input multiple-output (MIMO) wireless transceivers operating under digital beamforming architectures. Two distinct digital SIC approaches are evaluated, employing a generalized memory polynomial (GMP) model augmented with Itô–Hermite polynomial basis functions and a phase-normalized neural network (PNN) to effectively model the nonlinearities and memory effects introduced by transmitter and receiver hardware impairments. The robustness of the SIC is further evaluated under both single off-line training and closed-loop real-time adaptation, employing estimation techniques such as least squares (LS), least mean squares (LMS), and fast Kalman (FK) for model coefficient estimation. The performance of the proposed digital SIC techniques is evaluated through detailed simulations that incorporate realistic power amplifier (PA) characteristics, channel conditions, and high-order modulation schemes. Metrics such as error vector magnitude (EVM) and total bit error rate (BER) are used to assess the quality of the received signal after SIC under different signal-to-interference ratio (SIR) and signal-to-noise ratio (SNR) conditions. The results show that, for time-variant scenarios, a low-complexity adaptive SIC can be realized using a GMP model with FK parameter estimation. However, in time-invariant scenarios, an open-loop SIC approach based on PNN offers superior performance and maintains robustness across various modulation schemes.

## 1. Introduction

In modern wireless communication systems, the demand for high data rates and efficient spectrum utilization continues to grow exponentially, driven by the increasing scarcity of frequency resources needed to support emerging systems. With the rapid expansion of data-intensive applications such as 5G, 6G, IoT, and autonomous systems, ensuring seamless and reliable connectivity has become even more critical [1]. At the same time, minimizing latency is essential for real-time applications like autonomous vehicles, telemedicine, and extended reality, so latency can significantly impact performance and user experience. As wireless networks evolve to support these increasing demands, innovations in communication technology must prioritize both spectral efficiency and ultra-low-latency transmission to sustain the next generation of connectivity.

Despite the increasing demand for high-efficiency and low-latency communication, traditional wireless systems still rely on half-duplex transmission methods such as frequency division duplexing (FDD) and time division duplexing (TDD) [2], where devices send or receive data in separate time slots or use different frequency bands. While these methods may be adequate for certain use cases, they are less suited to modern applications [3].

**Figure 1:**
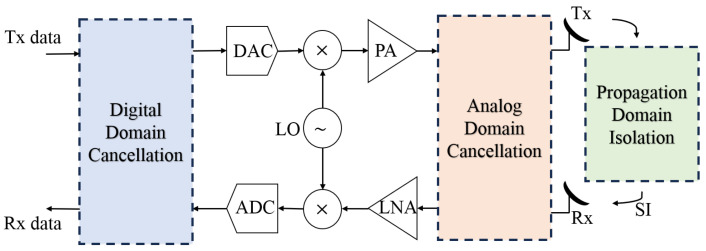
Model of full-duplex transceiver with passive and active SIC.

To overcome these issues, full-duplex communication, particularly IBFD, has emerged as a promising solution, enabling simultaneous transmission and reception on the same frequency, which is also known as simultaneously transmit and receive (STAR) systems [2]. This approach not only doubles spectral efficiency but also reduces latency. However, a major challenge in implementing IBFD systems is SI, where the transmitted signal leaks into the receiver path and interferes with the reception of much weaker incoming signals. This occurs because the transmitted signal, originating from a nearby antenna, can be up to 100 dB stronger than the desired signal arriving from a distant source [4]. To enable the successful deployment of IBFD technology, advanced SIC techniques are essential [5]. As can be seen in Figure 1, these SI cancellation techniques are generally classified into three main categories: passive suppression, active analog/RF cancellation, and digital/baseband cancellation [6].

Passive suppression is a technique that operates in the propagation domain, aiming to minimize SI power by leveraging advanced antenna technologies. This approach typically involves the use of several passive devices and techniques like circulators, highly directional antennas, adaptive beamforming, cross polarization, or absorptive shielding to reduce the power of the transmitted signal that impairs the receiver’s ability to detect the weaker incoming signal. As a result, passive suppression helps to decrease the SI at the source, before it even reaches the receiver [2,5].

In the RF/analog domain, active cancellation is typically implemented by injecting a cancellation signal that undergoes appropriate time delay, phase rotation, and attenuation to subtract a replica of the interfering transmitted signal from the received signal. This process helps mitigate SI before it reaches the receiver’s analog front-end, thereby preventing saturation. In some implementations, an additional transmitter chain is used to generate the cancellation signal [7,8]. Nevertheless, achieving perfect passive and analog/RF SI cancellation is both complex and expensive, making it difficult to eliminate SI entirely at the receiver. Even after the RF cancellation stage, a residual SI signal often persists due to transceiver imperfections that distort the SI waveform. These imperfections arise from various hardware impairments, such as nonlinearities in analog-to-digital converters (ADCs), in-phase/quadrature (I/Q) imbalance, and phase noise. In addition to the challenge of receiver desensitization, analog-domain SI processing becomes increasingly complex and less scalable in systems with a larger number of antennas, such as in MIMOconfigurations, where the cancellation of crosstalk self-interference between antenna elements becomes a critical design consideration [9,10]. Given these limitations, the focus is thus directed toward additionally canceling the SI in the digital domain, which serves as the final cancellation stage to suppress the residual SI that persists after propagation and analog suppression. This stage is essential for modeling hardware-induced nonlinearities and supporting adaptive, software-defined optimization, provided that the propagation and analog cancellation stages sufficiently suppress interference to maintain the receiver ADC’s required dynamic range [5].

In the digital/baseband domain, SIC is performed using sophisticated signal processing algorithms that subtract the residual interference after conversion to baseband, enabling complete removal of the SI component [11,12]. In some advanced systems, hybrid approaches are employed, integrating techniques from all three domains to achieve a higher level of SIC, such as zero-forcing beamforming [13], effectively suppresses linear SI in the RF/spatial domain. This highlights the importance of a hybrid SIC architecture that combines analog suppression with robust digital techniques to ensure reliable full-duplex operation even in harsh interference scenarios. To ensure optimal receiver performance, the SI must ultimately be canceled down to the receiver’s noise floor [14].

Recently, machine learning (ML)-based approaches have been increasingly explored for digital SI cancellation in FD and IBFD systems. In [15], a feed-forward neural network (FFNN) was employed to reconstruct SI signals in the digital domain, achieving superior cancellation performance compared to traditional polynomial models while maintaining lower computational complexity. More recent studies have investigated deep neural network architectures such as convolutional neural networks (CNNs) and gated recurrent units (GRUs), which enable effective modeling of both nonlinear and memory effects of the SI channel [16]. For instance, CNN–GRU hybrid models with residual and self-attention mechanisms have demonstrated up to 28–32 % improvements in nonlinear SIC capability compared to conventional adaptive and polynomial-based cancellers [16]. Moreover, in the context of IBFD communication networks, CNN-based methods have been applied to loopback channel estimation and denoising, outperforming classical Wiener filtering in both static and dynamic channel conditions [17]. Also, recent studies have begun integrating digital SIC with system-level AI frameworks, such as deep reinforcement learning (DRL), to jointly optimize interference management and energy efficiency. A DRL-based approach for energy efficiency maximization in RSMA-IRS-assisted ISAC systems was proposed in [18].

This paper investigates digital SIC using two distinct behavioral modeling approaches for SI characterization. First, a GMP model with Itô–Hermite nonlinear basis functions is employed, with coefficients estimated through LS, LMS, and FK algorithms to enable real-time adaptive operation. Second, for time-invariant scenarios, a PNN [19] is considered. The main contributions of this work are:

- Integration of the Itô–Hermite GMP model with adaptive estimation schemes (LS, LMS, FK) for robust, real-time digital SIC under nonlinear and time-varying conditions. To further enhance model efficiency, a doubly orthogonal matching pursuit (DOMP) algorithm is utilized for parameter reduction, effectively lowering computational complexity while preserving accuracy.
- Application and extension of the PNN framework for digital SIC, representing the first reported use of this approach in IBFD systems. The proposed PNN provides a compact and computationally efficient structure that eliminates the need for complex-valued processing through phase normalization, while preserving strong nonlinear modeling capability with substantially fewer parameters.
- Comprehensive performance evaluation in terms of error vector magnitude (EVM) and total bit error rate (BER) of the received signal after SIC, incorporating power amplifier (PA) nonlinearities, channel conditions, high-order modulation, and varying signal-to-interference (SIR) and signal-to-noise ratios (SNRs).

The remainder of this paper is structured as follows. Section 2 introduces a digital SIC architecture for IBFD systems, detailing the primary sources of SI across different transceiver configurations and outlining a digital SIC strategy integrated with MIMO beamforming and digital predistortion (DPD) techniques. Section 3 presents both the GMP model with Itô–Hermite polynomial basis functions and the PNN model, along with various coefficient estimation methods, including LS, LMS, and FK. Section 4 provides a comprehensive performance evaluation of the proposed models and solvers under realistic signal and hardware conditions, using EVM and BER metrics to assess their effectiveness across varying SIR and SNR scenarios. Finally, Section 5 concludes the paper.

## 2. Digital Self-Interference Cancellation in IBFD Communication Systems

**Figure 2:**
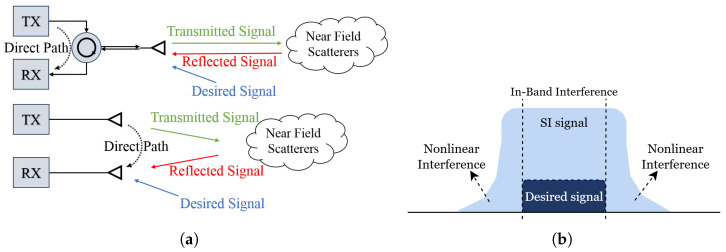
SI in IBFD systems: (**a**) Illustration of how SI arises from direct leakage, antenna coupling, and reflections in circulator-based and antenna-separation architectures [18]; (**b**) spectra showing the relative power levels of the desired and SI signals, demonstrating the dominance of SI in an IBFD scenario [6].

As highlighted in the previous section, a key challenge in realizing the spectral efficiency gains of IBFD communication is SI, where the transmitted signal contaminates the receiver, masking the desired signal. Figure 2a illustrates SI in two distinct IBFD configurations: one employing a circulator and the other utilizing separate antennas for transmission and reception. In the circulator-based approach, a single antenna is shared for both the transmitter (TX) and receiver (RX), with the circulator separating the transmitted and received signals. However, this setup fails to completely isolate the transmitted signal, resulting in SI from both direct leakage and reflections caused by near-field scatterers in the receiver. On the other hand, the antenna separation-based design mitigates direct leakage using dedicated TX and RX antennas, yet remains susceptible to SI due to signal coupling from the sidelobes of the TX’s antenna and environmental reflections [20]. As shown in Figure 2b, SI can severely degrade the desired communication signal, necessitating robust digital SIC techniques to effectively suppress interference and enable high-performance IBFD transmission [6].

**Figure 3:**
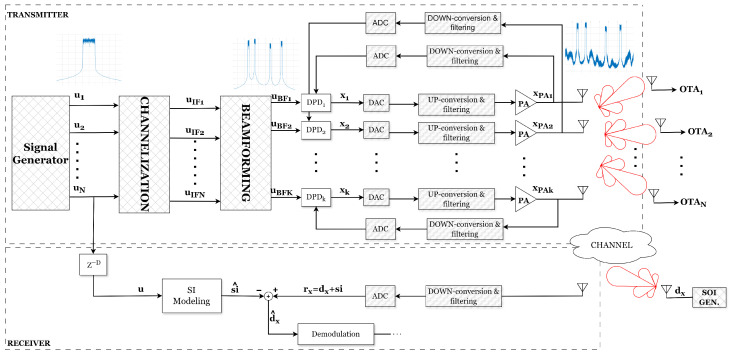
Blockdiagram of a MIMO transceiver with digital beamforming, DPD, and SIC, illustrating interference suppression and signal recovery in IBFD operation.

Figure 3 presents a simplified block diagram of a MIMO transceiver. The architecture consists of a signal generation block responsible for producing baseband waveforms, which are subsequently channelized to allocate them into *N* orthogonal frequency bands associated with *N* users. These signals are then processed by a beamforming stage, wherein spatial precoding is applied to steer the transmissions toward desired directions through *K* radiating elements, thereby synthesizing the corresponding transmit beams. However, one of the challenges associated with beamforming is the presence of sidelobes, which can introduce unwanted interference. In particular, these sidelobes can degrade receiver performance by causing desensitization, ultimately reducing the system’s ability to effectively process incoming signals. It should be emphasized that, at the receiver, the sidelobe components may exhibit power levels surpassing that of the desired signal, thereby potentially degrading link quality, as illustrated in Figure 2b.

To mitigate nonlinear effects and maintain signal integrity, DPD linearization is incorporated into the transmitter. DPD is applied at the baseband with the objective of linearizing the PAs in each transmit branch, thereby compensating for nonlinear distortions and improving the linearity of the transmitted signals. By incorporating DPD, the system is able to maintain the amplified signals with minimal distortion even when the PAs operate near their compression region, thus enhancing power efficiency and ensuring reliable performance in MIMO transmissions. This approach is especially critical for maintaining high spectral efficiency and minimizing interference, particularly in scenarios requiring precise beamforming to achieve optimal wireless communication performance.

However, a portion of the transmitted signal inevitably couples back into the receiver via antenna reflections, multipath propagation, or direct leakage, appearing as SI that can be suppressed in three different domains, as mentioned in Figure 1. In this work, to suppress the SI, a parallel digital SI cancellation path is implemented. As can be seen in Figure 3, this method begins by generating an estimate of the SI, $\hat{si}\left[n\right]$, based on a known version of the transmitted signal $u_{N}\left[n\right]$. After applying the appropriate delay alignment, $u\left[n\right]=u_{N}\left[n-D\right]$, the signal is processed through a nonlinear model designed to capture both linear and nonlinear impairments introduced by the DACs, upconverters, power amplifiers, analog front-end, and propagation channel, which collectively characterize the SI observed at the receiver.

As described in Figure 3, the received signal at RX, $r_{x}\left[n\right]$, comprises both the desired signal, $d_{x}\left[n\right]$, and the SI signal, si[n]. Therefore, once the SI has been estimated, i.e., $\hat{si}\left[n\right]$, it is subtracted from the received signal to extract the desired signal,

(1) $${\hat{d}}_{x}\left[n\right]=r_{x}\left[n\right]-\hat{si}\left[n\right]$$

with ${\hat{d}}_{x}\left[n\right]$ being the estimated desired signal.

## 3. Self-Interference Modeling

This section examines two alternative approaches for SI estimation: a GMP model employing Itô–Hermite polynomials, and an ANN-based framework. For the GMP-based approach, particular attention is given to the parameter extraction methodologies utilized.

### 3.1. Generalized Memory Polynomial Model with Itô–Hermite Polynomials

The modeling of SI is based on a nonlinear transformation of the delayed transmit signal u[n], which accounts for the effects of DACs, upconverters, PAs, analog front-end, and the wireless channel. The estimated SI signal $\hat{si}\left[n\right]$ is defined as:

(2) $$\hat{si}\left[n\right]=u\left[n\right]-v\left[n\right]$$

where v[n] represents the modeled distortion signal, capturing memory effects and nonlinearities in the system. The GMP model is used to model v[n], particularized with Itô-Hermite orthogonal polynomials [21] to provide an efficient basis for nonlinear expansion. The GMP model comprises three terms: the first captures direct memory effects, while the second and third account for lagging and leading cross-memory contributions, respectively. This can be expressed as:

(3) $$\begin{array}{cc} v[n] & =\sum_{l=0}^{L_{a}}\sum_{p=0}^{P_{a}}w_{p,l,m}^{a}\;u\left[n-l\right]\;\phi_{p}\left(|u\left[n-l\right]|\right)\\ \; & +\sum_{m=1}^{M_{b}}\sum_{l=0}^{L_{b}}\sum_{p=1}^{P_{b}}w_{p,l,m}^{b}\;u\left[n-l\right]\;\phi_{p}\left(|u\left[n-l-m\right]|\right)\\ \; & +\sum_{m=1}^{M_{c}}\sum_{l=0}^{L_{c}}\sum_{p=1}^{P_{c}}w_{p,l,m}^{c}\;u\left[n-l\right]\;\phi_{p}\left(|u\left[n-l+m\right]|\right) \end{array}$$

where u[n] and v[n] are the complex-valued input and modeled distortion signals, respectively. $P_{\left\{a,b,c\right\}}$ represents the polynomial orders, $L_{\left\{a,b,c\right\}}$ the memory depths, and $M_{\left\{b,c\right\}}$ the memory depths for the lagging and leading cross memory products. These parameters are selected to balance modeling accuracy with computational efficiency. Finally, $w_{p,l,m}^{\left\{a,b,c\right\}}$ are complex-valued parameters for specific values of *p*, *l* and *m*. The order *K* of the GMP model can be computed as $K=\left(P_{a}+1\right)\left(L_{a}+1\right)+P_{b}\left(L_{b}+1\right)M_{b}+P_{c}\left(L_{c}+1\right)M_{c}$.

In this paper, the nonlinear function $\phi_{p}\left(\cdot \right)$ represents an Itô–Hermite polynomial, providing an orthogonal representation of nonlinear dynamics for accurate and efficient modeling of SI in IBFD communication systems. These polynomials are orthogonal over the interval (−∞,∞) with respect to the Gaussian weight function. This orthogonality is particularly beneficial when the input signal is assumed to follow a Gaussian distribution, as it ensures that the basis functions are uncorrelated. Consequently, this facilitates a compact, numerically stable representation of nonlinear effects, which improves convergence and modeling accuracy. The standard Itô–Hermite polynomial used in this work is defined as:

(4) $$\phi_{p}\left(x\right)={\left(-1\right)}^{p}e^{x^{2}}\frac{d^{p}}{dx^{p}}e^{-x^{2}}\;$$

where *x* is identified as x=|u[n−l±m]| in (3). Alternatively, the classical polynomial basis can be expressed in the form,

(5) $$\phi_{p}\left(x\right)=x^{p}.$$

This conventional structure is commonly used in traditional memory polynomial models and does not inherently ensure orthogonality between terms. Although it offers a simpler implementation, this approach can result in reduced modeling efficiency and parameter coupling, particularly when the input signal follows a Gaussian distribution [22].

The optimal GMP configuration, defined by parameters such as polynomial order, memory depth, and cross-memory terms, is not known a priori. Using an excessively large parameter set increases complexity and may yield ill-conditioned models. To address this, the DOMP algorithm [23,24] is employed to select the most relevant basis functions, thereby reducing both the parameter count and the computational complexity of the GMP model.

The parameters of the GMP model can be estimated using several techniques. In this paper, we focus on three prominent methods: LS, LMS, and FK. Each of these approaches presents a distinct trade-off among estimation accuracy, computational complexity, and convergence behavior, making them suitable for different application scenarios depending on system constraints and performance requirements.

#### 3.1.1. Least Squares

The LS solution is obtained by jointly processing a set of input–output observation pairs; therefore, it can be regarded as a batch-based approach for estimating the SI signal. First, let us rewrite (2) in a more compact matrix notation,

(6) $$\hat{\mathrm{si}}=u-v=u-Uw$$

where $u\in C^{L\times 1}$ is the input vector and *L* is the number of samples (i.e., n=0,1,...,L−1); $\hat{\mathrm{si}}\in C^{L\times 1}$ is the estimated SI vector; and $w\in C^{K\times 1}$ is the vector of parameters, with *K* being the order of the model. The regression matrix $U\in C^{L\times K}$ containing the basis functions is defined as

(7) $$U={\left(\vartheta [0],\vartheta [1],\ldots ,\vartheta [n]\ldots ,\vartheta [L-1]\right)}^{T}$$

where $\vartheta \left[n\right]\in C^{K\times 1}$ is the vector of basis functions $\vartheta_{j}\left[n\right]$, for j=1,…,K, at time *n*.

(8) $$\vartheta^{T}\left[n\right]=\left(\vartheta_{1}\left[n\right],\vartheta_{2}\left[n\right],\ldots ,\vartheta_{j}\left[n\right],\ldots ,\vartheta_{K}\left[n\right]\right).$$

The basis functions can be constructed by particularizing them to any nonlinear behavioral model that admits a linear-in-parameters representation. If we now particularize using the GMP model in (3), the generic *j*th basis function at time *n* can be described as,

(9) $$\vartheta_{j}\left[n\right]=\vartheta_{p,l,m}\left[n\right]=u\left[n-l\right]\;\phi_{p}\left(|u[n-l\pm m]|\right)$$

with $\phi_{p}\left(\cdot \right)$ denoting the nonlinear function, which can be represented using polynomials as in (5) or Itô–Hermite polynomials as in (4).

Assuming that the SI signal, si[n] is not directly accessible, we use the received signal, $r_{x}\left[n\right]=d_{x}\left[n\right]+si\left[n\right]$, as a reference, under the assumption that the desired signal, $d_{x}\left[n\right]$, and the SI signal, si[n], are uncorrelated. The LS solution aims to find the vector of parameters $w\in C^{K\times 1}$ that minimizes the squared error between the received signal and the estimated SI. The cost function is defined as follows,

(10) $$J\left(w\right)={∥r_{x}-\;\hat{\mathrm{si}}∥}^{2}={∥r_{x}-\;\left(u-Uw\right)∥}^{2}.$$

Following a gradient descent approach, the parameters of the GMP-based SI model at the *i*th iteration can be calculated as follows,

(11) $$w^{i+1}=w^{i}-\mu \;\Delta w$$

with μ (0<μ<1) being a learning rate parameter. The LS solution for Δw is defined as

(12) $$\Delta w={\left(U^{H}U\right)}^{-1}U^{H}{\hat{d}}_{x}$$

where ^*H*^ denotes the conjugate transpose, and ${\hat{d}}_{x}\in C^{L\times 1}$ is the vector of the estimated desired signal, defined as

(13) $${\hat{d}}_{x}=r_{x}-\;\hat{\mathrm{si}}=r_{x}-\;\left(u-Uw\right).$$

Thus, the residual signal is driven toward the desired signal by estimating the SI component and subtracting it from the received signal [25].

#### 3.1.2. Least Mean Squares

The LMS algorithm is a widely used adaptive filtering method due to its simplicity, low computational cost, and effectiveness in tracking gradual variations in the SI channel [26]. In IBFD systems, LMS facilitates real-time adaptation to account for dynamic nonlinearities in the SI path caused by hardware imperfections, thermal drift, or nearby object movement. Unlike LS, the LMS algorithm is sample-oriented, and thus, the estimated SI is defined as

(14) $$\hat{si}\left[n\right]=u\left[n\right]-w^{H}\left[n\right]\;\vartheta \left[n\right]$$

where $\vartheta \left[n\right]\in C^{K\times 1}$ is the vector of basis functions as described in (8) and (9) and $w\left[n\right]\in C^{K\times 1}$ is the vector of parameters, both at time *n*.

Again, assuming that the desired signal, $d_{x}\left[n\right]$, and the SI signal, si[n], are uncorrelated. We made the independence assumption on purpose to focus only on how well the proposed algorithms model and adapt to nonlinear SI in the baseband domain. The estimation error is computed as

(15) $$e\left[n\right]={\hat{d}}_{x}\left[n\right]=r_{x}\left[n\right]-\hat{si}\left[n\right].$$

The parameters are estimated using the normalized LMS algorithm in an online (sample-by-sample) manner as follows,

(16) $$w\left[n+1\right]=w\left[n\right]+\mu \;e^{*}\left[n\right]\;\frac{\vartheta [n]}{\vartheta^{H}\left[n\right]\vartheta \left[n\right]+\varepsilon }$$

where * denotes conjugate, μ is the learning rate that controls the adaptation speed, and ε is a regularization term that improves numerical stability. While effective, LMS typically converges more slowly than second-order methods and requires careful selection of μ. A small μ ensures stability but slows adaptation, while a large μ accelerates convergence at the potential cost of instability or reduced accuracy in $\hat{si}\left[n\right]$ [27].

#### 3.1.3. Fast-Kalman

While LMS offers simplicity, it is often inadequate in situations requiring fast and accurate adaptation, especially in highly dynamic or rapidly changing SI environments. To address this, we adopt a FK filter-based algorithm, which leverages second-order statistical information to achieve superior convergence speed and tracking performance. Unlike the conventional Kalman filter, but like LMS, it does not require prior knowledge of a state transition matrix. The FK algorithm stems from the recursive least squares (RLS) family and dynamically updates both the SI model parameters and the inverse covariance of the estimation error. This allows it to adjust more aggressively and precisely in response to signal changes, making it suitable for systems with strong non-stationarities or nonlinearities.

Similarly to LMS, the model parameters are estimated using the FK algorithm in a sample-by-sample manner as follows,

(17) $$w\left[n+1\right]=w\left[n\right]+K\left[n\right]\;e^{*}\left[n\right]$$

where the estimation error e[n] is calculated as in (15) and where the Kalman gain $K\left[n\right]\in C^{K\times 1}$ is computed as,

(18) $$K\left[n\right]=\frac{P[n]\;\vartheta [n]}{Q_{M}+\vartheta^{H}\left[n\right]\;P\left[n\right]\;\vartheta \left[n\right]}$$

where $\vartheta \left[n\right]\in C^{K\times 1}$ is the vector of basis functions, $Q_{M}$ is the measurement noise scaling factor (e.g., $Q_{M}=0.99$) and $P\left[n\right]\in C^{K\times K}$ is the inverse error covariance [24] and it is updated as

(19) $$P\left[n+1\right]=P\left[n\right]-K\left[n\right]\;\vartheta^{H}\left[n\right]\;P\left[n\right]+Q_{P}$$

with the process noise matrix $Q_{P}\in C^{K\times K}$ typically chosen as a scalar multiple of the identity (e.g., $Q_{P}=10^{-16}I$), and the initialization of P[n] set as $P\left[n\right]=\delta^{-1}I$, with $\delta =10^{-14}$ [26,28].

FK adapts more aggressively than LMS and adjusts in real time based on the reliability of the new input. This makes it robust against non-stationarities and abrupt changes in the SI path. While FK is more computationally intensive, techniques like reduced-rank approximations can mitigate this cost, enabling deployment even in resource-constrained systems where high modeling accuracy is required.

### 3.2. Phase-Normalized Neural Network

In order to mitigate the inherent limitations in the modeling capabilities of approaches that rely on linear combinations of nonlinear basis functions, exemplified by the proposed GMP model, researchers have increasingly explored more sophisticated machine learning techniques. Among these, ANNs have gained particular attention due to their enhanced modeling power and ability to capture complex nonlinear relationships, thereby offering a promising alternative for digital SI reconstruction [15].

In this paper, we have considered an augmented real-valued time-delay feed-forward neural network that incorporates phase-normalization in the input layer [15]. This PNN has shown one of the best trade-offs between performance and model complexity in the field of PA modeling a DPD linearization [25,29].

**Figure 4:**
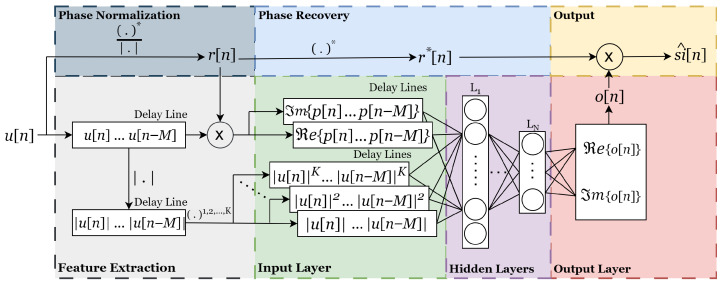
Representation of the PNN architecture.

The architecture of the PNN is illustrated in Figure 4. Feature extraction from the complex-valued input signal u[n] begins with generating the phase-normalized signal r[n] and conditioning the data for the input layer, which includes the incorporation of delay lines and power terms. The conditioned features are then provided to the input-layer neurons, taking into account both current and past samples of the real and imaginary components, as well as the associated power terms. These augmented terms at the input layer allow the network to learn both linear and nonlinear effects, crucial in IBFD systems with memory-dominant distortions. The principal learning capability of the ANN resides in the hidden layers, where the network weights are iteratively optimized to achieve the desired mapping at the output layer, o[n]. Finally, the output is denormalized, yielding $r^{*}\left[n\right]$, which is used to generate the output signal $\hat{s}i\left[n\right]$.

In contrast to the GMP model with real-time adaptation, the PNN is designed for time-invariant scenarios, where a single initial calibration is sufficient to ensure robust operation of the digital SIC. During the offline *factory calibration* stage, the PNN is trained to approximate the nonlinear SI path in an IBFD communication system. In this phase, the network learns the nonlinear mapping between the transmitted baseband signal and the corresponding SI observed at the receiver. For training purposes, it is assumed that the receiver measures only the SI component, i.e., $r_{x}\left[n\right]=si\left[n\right]$, without the presence of the desired signal $d_{x}\left[n\right]$.

The PNN is implemented as a fully connected feed-forward network with two hidden layers containing 20 and 10 neurons, respectively. This configuration was selected empirically to balance modeling capacity and training stability while avoiding overfitting. Nonlinear activation functions are used in the hidden layers, and a linear activation is applied at the output. Training is performed using the Levenberg–Marquardt (LM) algorithm, which offers fast convergence in nonlinear function approximation. The network minimizes the mean-squared error (MSE) between the modeled and measured SI signals:

(20) $$J\left[n\right]=\frac{1}{L}\sum_{n=1}^{L}{\left(si\left[n\right]-\hat{si}\left[n\right]\right)}^{2}.$$

The dataset is divided into 80% for training and 20% for validation. Early stopping based on the validation loss is applied to prevent overfitting and to ensure proper generalization. Each input vector consists of the real and imaginary components of the phase-normalized signal, defined as r[n]=u[n]/|u[n]|, along with their corresponding power terms up to order P=5 and memory depth τ=0:30. The network is trained using 400,000 samples and converges within 62 epochs (out of a maximum of 100) in approximately 110 min, with a minimum gradient threshold of $10^{-5}$. After convergence, the trained network parameters are stored and reused for subsequent evaluations without retraining, consistent with a time-invariant digital SIC scenario.

After the initial training, the ANN is used to estimate SI during subsequent iterations (where new data is generated) without further training,

(21) $$\hat{si}\left[n\right]=\mathrm{PNN}\left(u\left[n\right]\right).$$

The estimated SI is subsequently removed from the received signal, as expressed in (1), in order to isolate the desired signal and enhance its quality for subsequent processing, thereby improving overall detection performance.

## 4. Digital SIC Results

**Figure 5:**
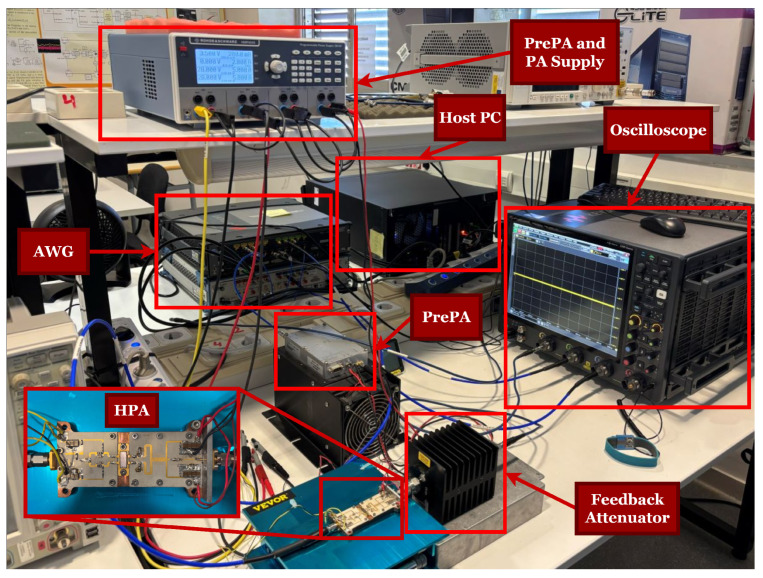
Experimental setup for data acquisition and validation of the proposed digital SIC techniques.

Digital SIC performance in IBFD systems is evaluated through simulations conducted in dynamic environments, including additive white Gaussian noise (AWGN) channels, as the objective is to isolate and evaluate the nonlinear and adaptive behavior of the proposed digital SIC algorithms. All simulations and signal processing procedures were implemented in MATLAB R2024a to ensure accurate modeling and analysis. To closely approximate real-world operating conditions, the nonlinear behavior of the transmitter’s power amplifier (PA) is modeled using black-box representations derived from measured input–output data. Figure 5 depicts the experimental configuration used for signal generation, transmission, and acquisition. The device under test (DUT) employs a broadband hybrid high-power amplifier (HPA) optimized for L-band applications, with its detailed specifications provided in [30].

DPD linearization is also considered in the transmitter in order to assess its influence on digital SIC in IBFD scenarios. The SI waveforms consist of 64-QAM OFDM signals with 100 MHz bandwidth, while the desired signal waveforms are *M*-QAM OFDM signals occupying 20 MHz, with M=16, 64, or 256. The simulator continuously generates data in batches of 1 ms at a clock rate of 500 MSa/s. Each new batch is processed as a single iteration, and the simulation proceeds over multiple iterations.

In this section, we assess the performance and limitations of digital SIC when employing both a GMP-based model and a PNN-based model to characterize the SI. For both methods, the estimated SI signal is subtracted from the received signal as depicted in Figure 3 and described in (1), and the resulting residual is used to recover the desired signal. The GMP model is implemented using Itô–Hermite polynomials, with coefficient extraction performed in either adaptive or non-adaptive mode. In the adaptive approach, the GMP parameters are continuously updated at each iteration, whereas in the non-adaptive approach, the parameters are computed only once, until convergence during the initial iteration, and then reused in all subsequent iterations. The parameters of the GMP model are estimated using a comparison of the LS, LMS, and FK algorithms.

**Figure 6:**
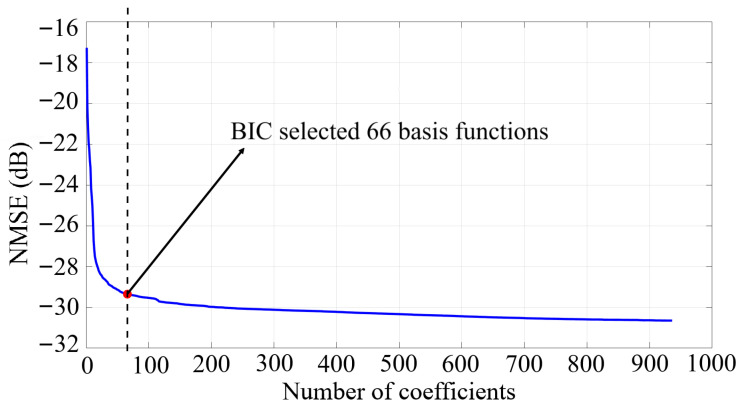
Impact of the DOMP algorithm on model performance.

The GMP formulation in (Section 2), includes three parts: the memory polynomial term and then, the lagging and leading cross-memory terms. The initial GMP model is parameterized by the polynomial orders $\;P_{a}=\left\{0:5\right\}$, $P_{b}=P_{c}=\left\{1:3\right\}$, with corresponding memory depths of $\;L_{a}=\left\{0:100\right\},L_{b}=L_{c}=\left\{0:5\right\}$. The lag and lead cross-terms are further controlled by shift indices $M_{b}=M_{c}=\left\{1:10\right\}$. This structure allows accurate modeling of both static and dynamic nonlinearities along the entire SI path. To limit the number of parameters and mitigate potential overfitting, the DOMP algorithm is combined with the Bayesian information criterion (BIC) to identify and retain only the most relevant basis functions [21]. As shown in Figure 6, out of the initial K=966 basis functions in the original parameter configuration, the BIC recommends using only the 66 most significant ones. This selection reduces dimensionality by more than 93 % while maintaining modeling accuracy. Consequently, the proposed approach supports an efficient and practical implementation of the SIC system with minimal performance loss.

Table 1 summarizes the parameter count and qualitative computational characteristics of the evaluated digital SIC algorithms. The LS approach employs 66 parameters and has the highest computational demand during the initial batch estimation, mainly due to the correlation and matrix inversion steps. After coefficient estimation, it operates with very low per-sample cost. The LMS algorithm also uses 66 parameters but performs simple iterative updates, making it the least complex and well suited for real-time adaptation, albeit with slower convergence. The FK method requires additional covariance storage $K^{2}$, resulting in 4422 effective parameters, but it achieves faster convergence and robust performance under time-varying conditions. Finally, the PNN contains 3341 trainable parameters across its layers and exhibits higher computational load only during offline training. Once trained, its inference stage is lightweight and comparable in complexity to LMS.

In order to evaluate digital SIC performance, we will evaluate the error vector magnitude (EVM) and bit error rate (BER) of the remaining desired signal after SI removal, i.e., ${\hat{d}}_{x}\left[n\right]$. The EVM is calculated as the root mean squared (RMS) value of the error normalized to a reference signal level (e.g., RMS of the ideal constellation) as follows,

(22) $$EVM=\frac{\sqrt{\frac{1}{N}\sum_{n=0}^{N-1}{\left(I_{meas}-I_{ref}\right)}^{2}+{\left(Q_{meas}-Q_{ref}\right)}^{2}}}{\sqrt{\frac{1}{N}\sum_{n=0}^{N-1}I_{ref}^{2}+Q_{ref}^{2}}}$$

where *N* represents the number of symbols, and *I* and *Q* denote the in-phase and quadrature components of a symbol, respectively. The subscripts ${\left(\cdot \right)}_{meas}$ and ${\left(\cdot \right)}_{ref}$ indicate the measured and the reference symbols, respectively.

In addition to EVM, which assesses symbol-level accuracy, we evaluate the system’s bit-level performance using the Total BER. This metric offers a comprehensive view of decoding reliability across all iterations, reflecting the overall effectiveness of each SIC method under dynamic signal conditions. The Total BER is calculated as

(23) $$\mathrm{Total}\;\mathrm{BER}=\frac{\sum_{i=1}^{N_{\mathrm{iter}}}E_{b}\left(i\right)}{N_{\mathrm{iter}}\times N_{b}}$$

where $N_{\mathrm{iter}}$ is the number of iterations, $E_{b}\left(i\right)$ denotes the number of erroneous bits in the *i*th iteration, and $N_{b}$ is the number of transmitted bits per iteration. This cumulative formulation captures bit-level performance over time, accounting for fluctuations in interference and channel conditions. The Total BER is computed per iteration and evaluated versus various signal-to-noise ratio (SNR) levels.

Besides SNR, another metric that significantly impacts the levels of SIC is the signal-to-interference ratio (SIR), which quantifies the ratio between the mean power of the desired signal and the mean power of the SI signal at the receiver—i.e., $\mathrm{SIR}\;\left(\mathrm{dB}\right)=P_{d_{x}}\;\left(\mathrm{dBm}\right)-P_{si}\;\left(\mathrm{dBm}\right)$.

**Figure 7:**
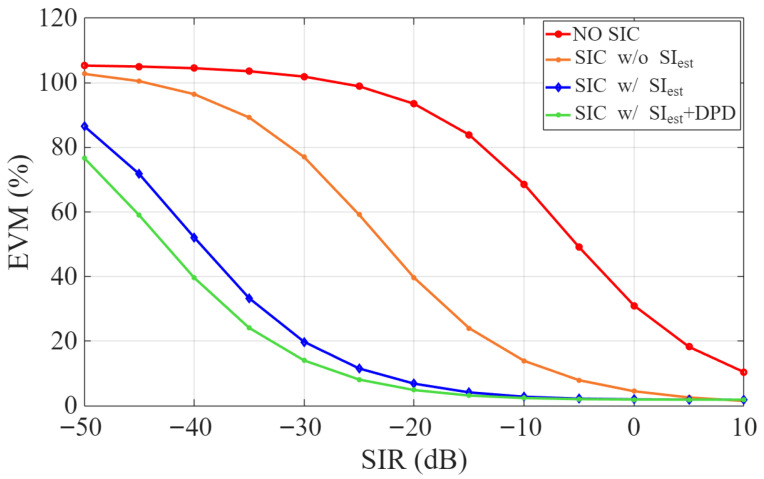
EVM vs. SIR for digital SIC configurations with and without SI estimation and DPD.

Figure 7 illustrates the EVM of the estimated desired signal, ${\hat{d}}_{x}\left[n\right]$, across different SIR values, considering four configurations: (i) no SIC, (ii) SIC without SI estimation (i.e., v[n]=0 in (2)), (iii) SIC with SI estimation, and (iv) SIC with both SI estimation and transmitter-side DPD. As expected, the EVM decreases with increasing SIR, underscoring the increased influence of the desired signal. At higher SIR levels (above 10 dB), the residual interference becomes negligible, which can limit the accuracy of SI estimation and slightly reduce cancellation effectiveness. Even without SI estimation, applying SIC yields a considerable improvement in EVM. Incorporating SI estimation yields further EVM improvement, while the combined use of SI estimation and DPD achieves the greatest reduction. These findings demonstrate the effectiveness of digital SIC, particularly when combined with accurate SI estimation and transmitter linearization, in suppressing residual interference and preserving the desired signal integrity. However, under very low SIR conditions (below –30 dB), further suppression using analog techniques and propagation-domain isolation is required to reduce residual SI to levels manageable by digital cancellation.

**Figure 8:**
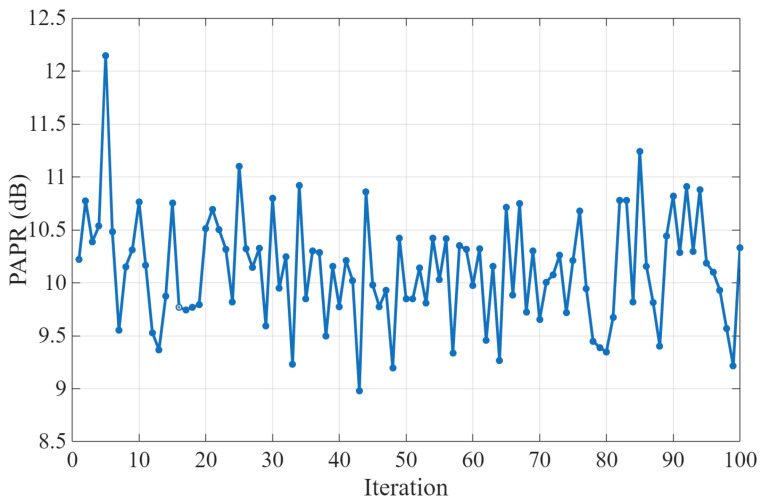
PAPR of the transmitted signal across iterations.

To assess the robustness of SI estimation, we evaluate SIC performance over time by running up to 100 iterations with different data batches for both the transmitted and desired signals. As illustrated in Figure 8, the peak-to-average power ratio (PAPR) varies across iterations for the 64-QAM OFDM transmitted signals with 100 MHz bandwidth. A comparable behavior is observed for the 16-QAM OFDM desired signal with 20 MHz bandwidth.

**Figure 9:**
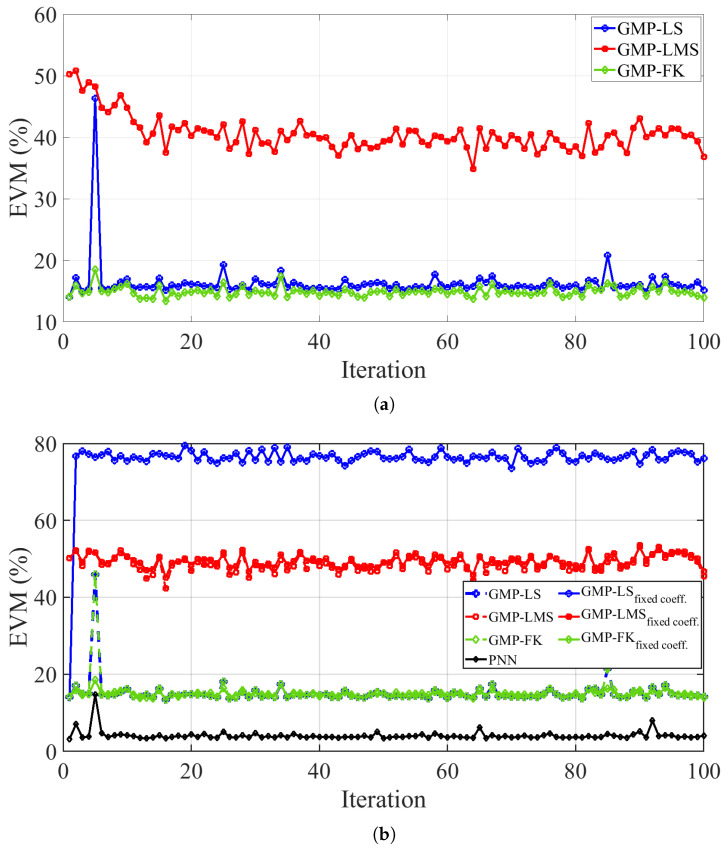
EVM across iterations evaluated under (**a**) the adaptive GMP model with various parameter estimation algorithms, and (**b**) the non-adaptive GMP and PNN models trained offline using different parameter estimation algorithms.

Figure 9 presents the EVM of the recovered desired signal across iterations for a SIR of 30 dB. In Figure 9a, the GMP model using Itô–Hermite polynomials is considered, with coefficients continuously adapted via LS, LMS, or FK. In contrast, Figure 9b shows the case where the coefficients are not continuously updated; they are computed only once at the first iteration and then reused for all subsequent iterations. In addition to the GMP model with different coefficient adaptation strategies, the PNN model is also included for comparison. As observed, the non-adaptive approach yields robust results, with EVM values remaining largely consistent across iterations.

As shown in Figure 9a, the FK method consistently achieves the lowest EVM values among the traditional approaches, indicating superior accuracy and robustness throughout iterations. Its rapid convergence and stability are attributed to the adaptive nature of the Kalman filtering process, which leverages the Kalman gain to dynamically weigh new observations based on their estimated reliability. This enables real-time model adjustments in response to changes in the interference path. In comparison, the LMS method maintains the highest EVM values across iterations and reflecting slower convergence. The batch-oriented LS method performs well, with relatively stable EVM levels but not matching the robustness or accuracy obtained with FK. Overall, FK demonstrates the best trade-off between convergence speed and resilience among the traditional methods.

Moreover, as shown in Figure 9b, the PNN model demonstrates superior modeling performance, and consequently better SIC, compared to the GMP model (independent on the parameter identification method), consistently achieving the lowest EVM, with values oscillating around 4% across all iterations. It is noteworthy that when the GMP coefficients are trained offline using LS, the solution exhibits poor robustness, with the EVM increasing sharply to about 76%. In contrast, for the LMS and FK algorithms, when comparing online adaptation with no adaptation (i.e., only online training), the results show no significant difference. In general, the EVM values remain fairly stable across iterations. However, at certain iterations (e.g., iteration 5), a noticeable degradation can occur. As shown in Figure 8, these EVM spikes coincide with a pronounced increase in PAPR. This correlation indicates that nonlinear distortion from the transmitter’s PA may have accentuated the residual SI, thereby reducing the effectiveness of digital cancellation in that iteration.

**Figure 10:**
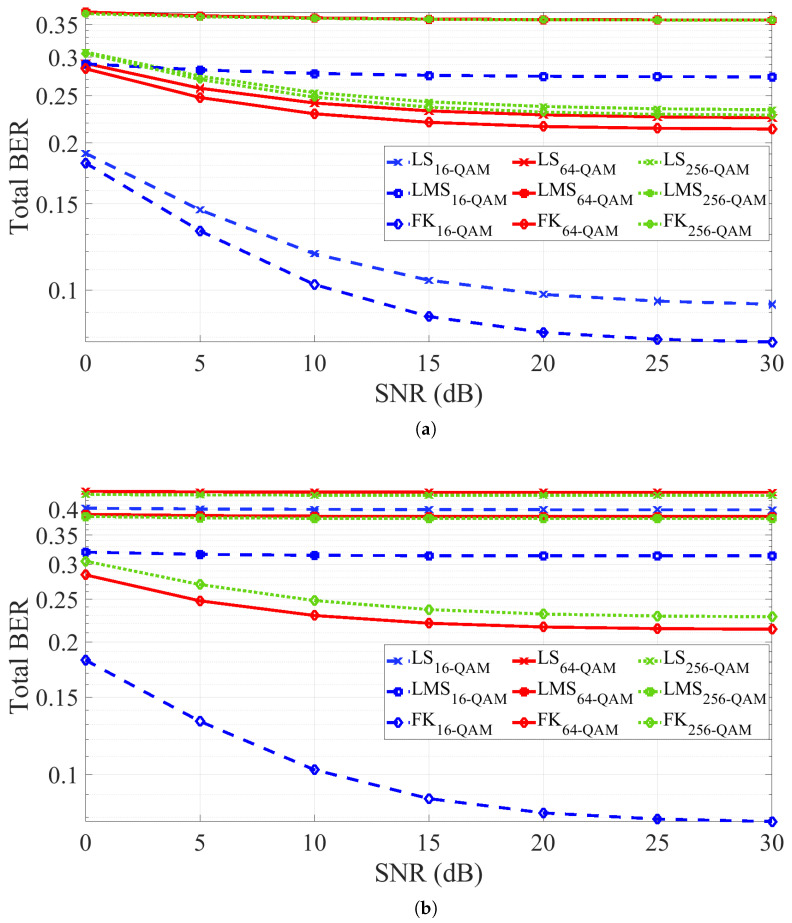
BER performance of the GMP-based SIC with SIR=30 dB across SNR values and different modulation formats: (**a**) with parameters adaptation, and (**b**) without parameters adaptation.

To evaluate the robustness of the GMP-based SIC under a SIR of 30 dB, the BER performance is assessed across different SNR values using the GMP model with both adaptive (Figure 10a) and non-adaptive (Figure 10b) configurations. The analysis covers three modulation schemes: 16-QAM, 64-QAM, and 256-QAM. Although the EVM compensation after SIC is comparable, the BER varies significantly with the modulation order. As expected, higher-order modulations yield higher BER, particularly at low SNR values. Figure 10a,b shows that the FK method consistently achieves the lowest BER, even without adaptation, highlighting its strong adaptive properties and robustness, consistent with the earlier EVM analysis. Conversely, in the absence of adaptation, the GMP model with LS estimation exhibits a marked degradation in performance, in line with the EVM results previously discussed.

**Figure 11:**
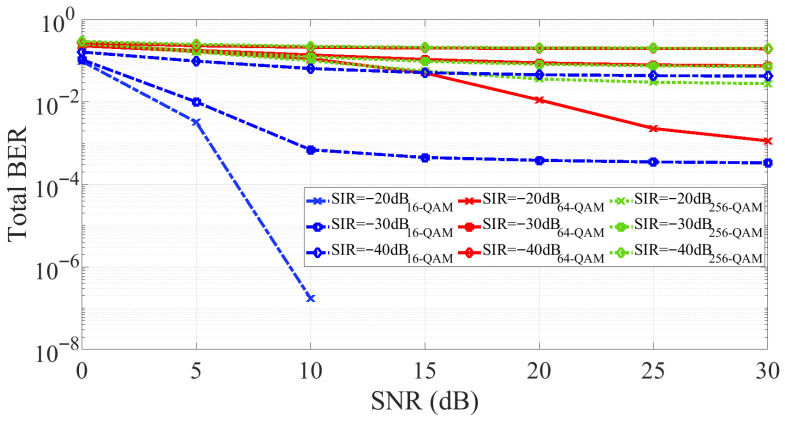
BER performance of the PNN-based SIC across SNR values and different modulation formats and SIR values.

Figure 11 illustrates the BER performance of the PNN-based SIC across different SNR values for various modulation schemes and SIRs. Once the PNN is trained offline for a given SIR and modulation scheme, it is not re-trained for different SNR conditions. As in Figure 10, higher-order modulations result in higher BER, while increasing the SIR improves BER. However, also consistent with Figure 10, the BER improvement with increasing SNR saturates beyond a threshold of approximately 15 dB, after which the SIR becomes the dominant factor. Notably, the PNN-based SIC consistently outperforms the GMP-based SIC at low SNR values. For example, at an SIR of −30 dB and SNR of 10 dB, the PNN-based SIC achieves a BER below $10^{-3}$ with 16-QAM, whereas the GMP-based SIC with FK adaptation yields a BER of about $10^{-1}$ under the same conditions. The PNN-based SIC method demonstrates strong generalization across different modulation formats and interference levels, confirming its potential as a non-adaptive, data-driven solution for IBFD communication systems.

**Figure 12:**
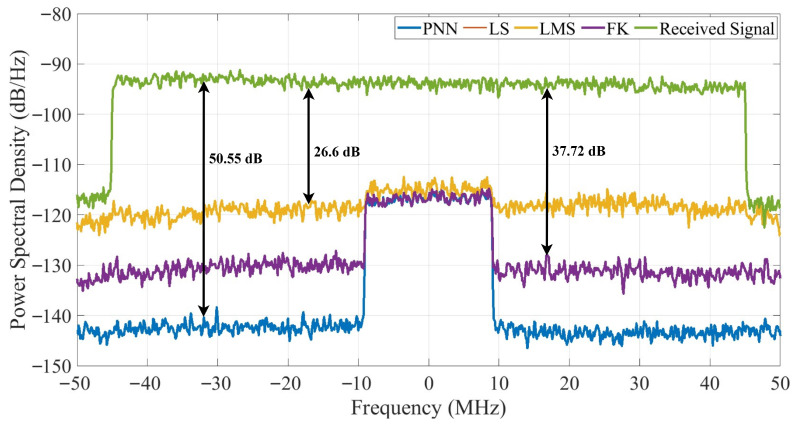
Comparison of the power spectral density of various digital SI cancelers considering SIR = −30 dB and SNR = 30 dB.

Figure 12 shows the power spectral density (PSD) of the received and residual SI signals after digital cancellation, assuming that the propagation and analog domains have already provided initial suppression. The obtained residual SI suppression levels are approximately 50.55 dB for PNN, 37.72 dB for FK and LS, which exhibit nearly identical performance, and 26.6 dB for LMS, when SIR = −30 dB and SNR = 30 dB, indicating that the proposed PNN achieves the most effective nonlinear cancellation. These results confirm that, once the analog and propagation domains have reduced the SI to manageable levels, digital-domain cancellation can further suppress the remaining components and achieve near-complete mitigation in IBFD systems.

## 5. Conclusions

This study has presented a comprehensive evaluation of digital SIC strategies for IBFD communication systems, comparing a GMP model with Itô–Hermite polynomials and a PNN. Model order reduction through the DOMP algorithm effectively minimized the number of basis functions in the GMP structure, while parameter identification using the FK and LMS algorithms enabled adaptive operation and was benchmarked against the conventional LS approach. Extensive simulation studies evaluated post-SIC performance in terms of EVM and BER under varying SIR and SNR conditions, data types, and modulation formats. The results show that a low-complexity adaptive SIC can be achieved with the FK-based GMP, which consistently outperformed LS and LMS in dynamic scenarios. In parallel, ANN-based approaches, such as the proposed PNN, demonstrated the best steady-state performance for static conditions, maintaining robust cancellation without online adaptation. Integrating DPD at the transmitter further improved overall SIC efficiency, particularly under strong interference. Although lower-order modulations benefited substantially from digital SIC, higher-order constellations such as 256-QAM remained sensitive to residual interference, underscoring the importance of complementary suppression in the analog and propagation domains. Overall, the findings highlight that FK-based GMP and PNN techniques offer distinct yet complementary trade-offs between adaptability, complexity, and steady-state accuracy, enabling efficient and scenario-dependent SIC solutions for next-generation IBFD transceivers.

From an implementation standpoint, the GMP model with FK-based adaptive parameter identification offers a viable low-complexity solution for DSP deployment. In contrast, the PNN still demands further optimization to reduce computational load and enable real-time adaptive training, facilitating practical integration in IBFD systems.

Future work will focus on experimental validation using a real IBFD transceiver testbed to assess robustness against hardware impairments such as phase noise, IQ imbalance, and quantization effects. The real-time feasibility of the proposed digital SIC techniques or semi-supervised retraining schemes in ML-based methods will also be explored through DSP and FPGA implementation, leveraging model pruning, coefficient quantization, and reduced-rank representations to enhance efficiency. Furthermore, the comparative framework can be extended for SDR-based evaluation, providing valuable insight into performance–complexity trade-offs and hardware adaptability in next-generation FD systems.

## Figures and Tables

**Table 1:** Comparison of parameter count and computational complexity for various digital SIC methods.

Model	Adaptation Method	Parameter Count	Comments
GMP (Itô–Hermite)	LS	K = 66	High complexity, non-adaptive.
GMP (Itô–Hermite)	LMS	K = 66	Low complexity, slower convergence.
GMP (Itô–Hermite)	FK	K + $\left(K^{2}\right)$ ^#^ = 4422	Fast convergence and robust performance in dynamic conditions.
PNN	LM	$P=\sum_{l=1}^{L}M_{l}\left(N_{l}+1\right)$ = 3341 *	Highest accuracy, trained once.

Notes: ^#^ Extra storage required for the FK covariance. * *M* and *N* denote the number of neurons in each layer and the number of input neurons, respectively. *L* denotes the number of layers in the neural network (excluding the input layer).

## Data Availability

The data presented in this study are available on reasonable request from the corresponding author.
